# A new digital biomarker of *Demodex* blepharitis: energy curve of the meibomian edge

**DOI:** 10.3389/fcell.2025.1627327

**Published:** 2025-08-05

**Authors:** Minjia Wang, Xiaoyu Chen, Kesheng Wang, Kunhui Xu, Xinxin Yu, Qi Dai, Min Ren

**Affiliations:** ^1^ Zhejiang Hospital, Hangzhou, China; ^2^ Eye Hospital, Wenzhou Medical University, National Clinical Research Center for Ocular Diseases, Wenzhou, China; ^3^ College of Mathematical Medicine, Zhejiang Normal University, Jinhua, China

**Keywords:** artificial intelligence, *Demodex* blepharitis, digital biomarker, energy curve, uneven atrophy

## Abstract

**Purpose:**

To develop and validate a novel digital biomarker, the energy curve of the meibomian gland (MG) edge, to assess MG uneven atrophy and aid in diagnosing *Demodex* blepharitis.

**Methods:**

A retrospective study enrolled 76 dry eye patients (42 with *Demodex* blepharitis, 34 controls). Segmentation of upper eyelid meibography images was accomplished via a convolutional neural network (CNN)-based artificial intelligence (AI) model. The lower margin curve of MGs was extracted using an active contour model (Snake) to compute a composite energy value that integrates elastic, curvature, and smoothness energies. Clinical parameters, including non-invasive tear breakup time (NIBUT), lid margin score, and Meiboscore, were evaluated.

**Results:**

The *Demodex* group showed shorter NIBUT (median: 2.84 vs. 5.18 s, *p* < 0.001) and higher lid margin scores (median: 2 vs. 1, *p* = 0.002) and Meiboscores (median: 1 vs. 1, *p* = 0.009). The *Demodex* group also exhibited significantly higher energy curve values than controls (median: 32.44 vs. 11.20, *p* < 0.001), reflecting pronounced uneven gland atrophy. Meanwhile, MG density significantly influenced energy curve values (*p* = 0.010). After adjusting for MG density, the energy curve demonstrated strong diagnostic accuracy (AUC = 0.897, sensitivity 78.6%, specificity 91.2%).

**Conclusion:**

The energy curve quantifies structural irregularities in MGs caused by *Demodex* infestation, serving as a non-invasive biomarker for early diagnosis. Its integration with meibography enhances clinical workflows, particularly in resource-limited settings.

## 1 Introduction

*Demodex* mites are among the most common human parasites, including only two species: *Demodex folliculorum* and *Demodex brevis*. *Demodex folliculorum* is mostly parasitic on eyelash follicles, while *D. brevis* predominantly inhabits sebaceous glands and meibomian glands (MGs). *Demodex folliculorum* was first identified in human hosts in 1841, with subsequent confirmation of its presence within the MG ducts in 1875 (1). Furthermore, the prevalence of *Demodex* infestation demonstrated a significant upward trend with advancing age, peaking in the fifth and sixth decades of life. The infection rate among children is low, at only 13% among those aged 3-15. In contrast, epidemiological data show that the infection rate among people aged 31-50 is 69%, and it approaches 95%–100% in individuals over 70 years (Elston and Elston, 2014; Cheng et al., 2015; Bitton and Aumond, 2021).

Ocular *Demodex* infestation represents a highly prevalent yet overlooked cause of ocular surface inflammation that contributes to multiple ocular surface disorders, including blepharitis, meibomian gland dysfunction (MGD), recurrent chalazia, and keratoconjunctivitis (Kim et al., 2011; Akkucuk et al., 2023; Yan et al., 2020). *Demodex* infestation contributes to ocular surface disease mainly through the following mechanisms. The mites can obstruct the sebaceous gland or the MG ducts through mechanical blockage. *Demodex* mites can also serve as microbial vectors, contributing to the occurrence of disease. Studies have confirmed that *D. folliculorum* can carry a variety of pathogens such as bacteria, fungi and viruses. Moreover, the mite’s cytoskeleton may serve as a foreign body to trigger a granulomatous reaction, similar to the pathogenesis of chalazion, or stimulate inflammatory and immune responses (Rabensteiner et al., 2019; Cheng et al., 2015; Deng et al., 2024).

MGD secondary to *Demodex* infestation arises from mechanical ductal occlusion and subsequent inflammatory cascades, and a higher density of *Demodex* infestation exhibits a positive correlation with the degree of structural and functional deterioration in MGs (Yeu and Koetting, 2025). Liang et al. (2018) demonstrated that *D. brevis* was associated with severe MGD, characterized by the loss of more than one-third of the MGs in a young patient population. Studies demonstrated that *Demodex* infestation altered MGs microstructure, including acinar dimensions, fibrosis severity, and meibum reflectivity, with more pronounced effects observed in patients with MGD (4). Moreover, MGs morphological change, mainly including MGs loss or dropout, is associated with the function of MGs (Ding et al., 2024). Clinically, we have observed uneven atrophy of MGs in patients with mite infection. Yin and Gong (2015) also confirmed that uneven atrophy of the MGs did exist, and the nasal segment dropout contributed more significantly than other dropout regions to the development of MGD. Our previous study also demonstrated that *Demodex* mite infestation could cause uneven gland atrophy and proposed some novel parameters to evaluate uneven atrophy of MGs (Liu et al., 2022; Yu et al., 2022). However, current research on uneven MG atrophy remains limited. There is a lack of effective parameters to accurately assess uneven MGs atrophy in very severe or very mild MGs atrophy. The relationship between uneven gland atrophy and the function of MGs remains unclear. Furthermore, the mechanism of uneven atrophy of MGs caused by *Demodex* mites is not clear, and further research is needed to explore this.

The energy of a curve is a mathematical tool employed to quantify the shape and characteristics of a curve. A wide range of fields have extensively utilized it, such as computer-aided design (CAD), computer graphics, image processing, and differential geometry. Its applications in medicine are extensive and significant, particularly in the fields of image segmentation (Pamela and Kashyap, 2016) and dual-energy computed tomography (CT) (Lu et al., 2021). By analyzing the attenuation characteristics of rays or tissues, it provides a powerful tool for lesion detection, disease assessment, and monitoring of therapeutic efficacy.

The uneven atrophy of MGs can be reflected in the undulations of the fitted curve of the gland’s lower margin. This links the unevenness to the curve’s morphology. Therefore, we extracted the lower margin curve of the MG and analyzed it using curve energy. This allowed us to investigate whether the characteristics of uneven MG atrophy could serve as an auxiliary diagnostic tool.

## 2 Materials and methods

### 2.1 Participants

We conducted a retrospective study and recruited patients diagnosed with dry eye at the Eye Hospital, Wenzhou Medical University, from February 2024 to April 2024. A total of 76 patients were included in this study, all of whom accepted a comprehensive and meticulous examination by professional ophthalmologists using a slit lamp and were diagnosed with dry eye syndrome. Moreover, all patients underwent eyelash epilation for microscopic examination to detect *Demodex* mite infestation. Of these patients, 42 were diagnosed with *Demodex* blepharitis, while 34 were found to be free from mite infection. Exclusion criteria encompassed prior ocular trauma/surgery, active ocular surface inflammation, contact lens use within 14 days, tear film-altering medications, and systemic conditions affecting ocular surface homeostasis. To minimize research errors, patients with near-complete atrophy of the MGs were not included. Data from only the right eye was included in the analysis. This study adhered to the Declaration of Helsinki principles and received approval from the Research Ethics Committee of the Affiliated Eye Hospital of Wenzhou Medical University (approval number: 2023-022-K-120).

### 2.2 Diagnosis of *Demodex* blepharitis

The etiological diagnosis of *Demodex* mites relies on laboratory examination (Lin et al., 2024). For microscopic examination, three eyelashes are taken from each eyelid, preferably those with cylindrical dandruff or those with inverted or misaligned eyelashes.

Epilated eyelashes were aligned longitudinally on glass slides, fixed with a coverslip, and subjected to light microscopy for mite quantification. Adding 20 μL of cedar oil and 100% ethanol or 0.25% sodium fluorescein solution to the slide can enhance the visibility of the mites (Branch, 2018).

The diagnosis of *Demodex* blepharitis is based on the following criteria (Branch, 2018): (Rabensteiner et al., 2019) Chronic or subacute course in both eyes, with symptoms such as redness, itching, and foreign body sensation, or accompanied by recurrent and refractory chalazion; (Elston and Elston, 2014); Abnormal eyelashes with cylindrical dandruff (which have diagnostic value), and probably accompanied by lid margin congestion and hypertrophy; (Cheng et al., 2015); Positive detection of *Demodex* mites. A definitive diagnosis of *Demodex* blepharitis can be made if all three criteria are met. However, without clinical symptoms or signs, the presence of *Demodex* mites alone can not be diagnosed as *Demodex* blepharitis.

### 2.3 Ocular surface evaluation

All patients received comprehensive ocular surface evaluations performed by professional ophthalmologists. The objective assessments included tear meniscus height (TMH), non-invasive breakup time (NIBUT), corneal fluorescein staining (CFS), lid margin abnormality score, and Meiboscore. Additionally, all patients filled out the Ocular Surface Disease Index (OSDI) questionnaire, providing detailed insights into their symptoms. The TMH was measured by Keratograph 5M (K5M; Oculus, Wetzlar, Germany), which also enabled automated analysis of NIBUT. CFS was assessed following the application of fluorescein dye. Lid margin abnormalities were evaluated using the Baylor grading scheme, which includes parameters such as anterior or posterior displacement of the mucocutaneous junction, vascular engorgement, obstruction of MG orifices, and irregularity of the lid margin. Meibography images were obtained, and scoring was performed based on the extent of MG dropout. The OSDI questionnaire was used to comprehensively assess the impact of ocular surface disease on patients’ quality of life by evaluating the frequency and severity of dry eye symptoms, their influence on daily activities, and the discomfort experienced in specific environments.

### 2.4 Image acquisition and meibomian gland segmentation

Meibography images were captured by Keratograph 5M (K5M; Oculus, Wetzlar, Germany). Images that were blurred and had indistinct structures would be excluded. Prior to this, our team had already developed an AI model for MG extraction based on Convolutional Neural Networks (CNN) (Dai QL et al., 2021). The model demonstrated a high performance with an intersection over union (IoU) of 0.9077% and 100% repeatability. It could effectively segment MGs and the tarsus from meibography images, and generate segmented images of individual glands and the tarsus. Additionally, it enabled the precise calculation of various morphological parameters, including MG density (Zhang et al., 2022) and vagueness.

### 2.5 Energy curve

**FIGURE 1:**
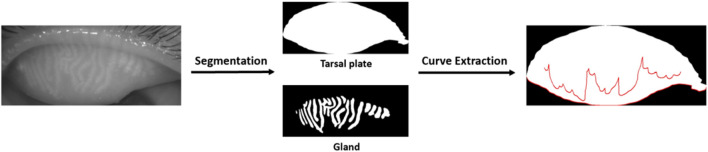
Meibomian gland segmentation and curve extraction.

The edge curve of the MG was extracted using the Snake model (Active Contour Model, ACM) based on the segmented glands. The Snake model evolved an initial contour within the image iteratively until it conformed to the target boundary (Figure 1). This process was driven by the minimization of the curve energy, which could be represented as:

$$E=\int E_{internal}+E_{image}+E_{constraubt}\;ds$$

In this equation, $E_{internal}=\varphi {\left|v^{\prime }\left(s\right)\right|}^{2}+\eta {\left|v^{\prime \prime }\left(s\right)\right|}^{2}$ represents the internal energy of the curve, φ controls the elasticity of the curve, and η governs the smoothness of curvature. $E_{image}=-\left|\nabla I\left(x,y\right)\right|$ , $I\left(x,y\right)$ denotes the image grayscale value, while the gradient ∇I attracts the Snake curve towards the boundary of the MG. The curve evolves continuously through gradient descent, and when the energy function reaches its minimum value, the resulting curve corresponds to the lower margin curve of the MG.

Informed by the principles of the Snake model, we introduced a method to quantitatively evaluate the unevenness of MG atrophy through the calculation of curve energy. This approach involves modifying the energy terms to extract the internal energy value of the curve. The expression is as follows:

$$E_{total}=\int E_{elasticity}+E_{curvature}+E_{smoothness}\;ds$$

In this equation, $E_{elasticity}$ denotes the elastic energy:

$$E_{elasticity}\frac{\alpha }{L}\int \left|v^{\prime }{\left(s\right)}^{2}\right|\;ds$$

$E_{curvature}$ represents the curvature energy:

$$E_{curvature}=\frac{\beta }{L}\int \kappa \left(s\right)\;ds$$

$E_{smoothness}$ signifies the smoothness energy:

$$E_{smoothness}=\frac{\gamma }{L}\int \left|v^{\prime \prime }{\left(s\right)}^{2}\right|\;ds$$

$L=x_{\max}-x_{\min}$ indicates the horizontal span of the curve. α , β , γ are the weighting factors, each with a default value of 1. $\kappa \left(x\right)$ represents the curvature, calculated as follows:

$$\kappa \left(x\right)=\frac{\left|y^{\prime \prime }\left(x\right)\right|}{{\left(1+{\left(y^{\prime }\left(x\right)\right)}^{2}\right)}^{\frac{3}{2}}}$$

The elastic energy quantifies the local elastic variations of the curve, while the curvature energy reflects the overall degree of bending. The smoothness energy signifies the curve’s smoothness characteristics.

In this study, the three principal weighting factors of the Snake model were set to α=0.1 , β=1.0 , and γ=0.1 . This parameter configuration provided favorable contour fitting and boundary smoothness in the experimental images. The parameter selection was based on considerations of edge strength, target complexity, model stability, and empirical observations. The convergence criteria were defined as a maximum contour point displacement of less than 0.5 pixels or a total energy change below 10^–4^, with the maximum number of iterations set to 5000. Among these parameters, γ exhibited a significant influence on the energy value and the contour adherence to object boundaries. β controlled the smoothness of the contour, while α regulated the contraction tendency of the curve. These three parameters must be jointly adjusted to achieve optimal performance.

### 2.6 Statistical analysis

Data analysis was performed with SPSS (version 25.0). The Kolmogorov-Smirnov test was employed to evaluate the normality of all datasets. Differences between patients with *Demodex* blepharitis and negative control subjects were evaluated using either the independent samples t-test or the Mann-Whitney U test. A two-sided *p* < 0.05 was considered statistically significant.

## 3 Results

### 3.1 Ocular surface examination

In this study, the examination information of 76 patients was collected. Of these, 42 were diagnosed with *Demodex* blepharitis, and 34 patients with dry eye disease but without *Demodex* blepharitis were enrolled as the control group. The basic characteristics and clinical parameters of the participants are shown in Table 1. No significant difference was found in TMH, CFS, or OSDI between the two groups. However, significant differences were observed in NIBUT, lid margin score, and meiboscore between the two groups (all *p* < 0.05).

**TABLE 1:** Basic characteristics and clinical parameters.

Clinical characteristics	*Demodex* group	Control group	p
Age	37.238 ± 11.603	33.500 (22.750,48.250)	0.758
TMH	0.220 (0.190,0.250)	0.215 (0.190,0.243)	0.416
NIBUT	2.840 (2.00,3.015)	5.175 (2.680,10.273)	<0.001
CFS	0 (0,2)	0 (0,1.25)	0.737
Lid margin score	2 (1,3)	1 (0,2)	0.002
Meiboscore	1 (1,2)	1 (1,1)	0.009
OSDI	22.920 (13.540,39.585)	8.330 (2.000,39.205)	0.071

### 3.2 Curve energy

We calculated the curve energy values for both the MG and the tarsus margin. According to the research objective, the MG curve energy value was defined as the raw value. Then, we corrected this raw value considering the influence of tarsus morphology on MG morphology. The difference, obtained by subtracting the tarsus margin curve energy value from the raw value, was used as a corrective index. Table 2 shows the comparison of the curve energy values and the corrective index between the two groups, revealing significant differences (all *p* < 0.001).

**TABLE 2:** Curve energy values.

Energy indices	*Demodex* group	Control group	p
Curve Energy	32.444 (21.063,56.394)	11.195 (5.251,19.260)	<0.001
Curve Energy ^correct^	32.068 (20.618,56.115)	10.410 (4.649,18.600)	<0.001

### 3.3 Meibomian gland morphological parameters

Considering that the fluctuation of the curve may be influenced by the parameters of the MGs, we employed multivariate regression analysis to investigate the impact of various parameters. The results are shown in Table 3. Our analysis revealed that among these parameters, MG density has a significant impact on curve energy and corrected values (p < 0.05). So, we adjusted the raw values by incorporating this key factor. Specifically, we divided the above curve energy by the density to ensure the accuracy and reliability of the results.

**TABLE 3:** Multivariate linear regression.

Clinical characteristics	Curve energy		Curve energy ^correct^	
	Coefficient	p	Coefficient	p
NIBUT	−0.878	0.195	−0.906	0.182
Lid margin score	−1.015	0.691	−1.000	0.696
Meiboscore	3.373	0.491	3.417	0.486
MG number	0.888	0.379	0.886	0.380
Average MG area	0.004	0.379	0.004	0.386
MG density	−212.706	0.010	−211.329	0.011
MG vagueness	−0.699	0.136	−0.698	0.137

### 3.4 Receiver operating characteristic curves

**FIGURE 2:**
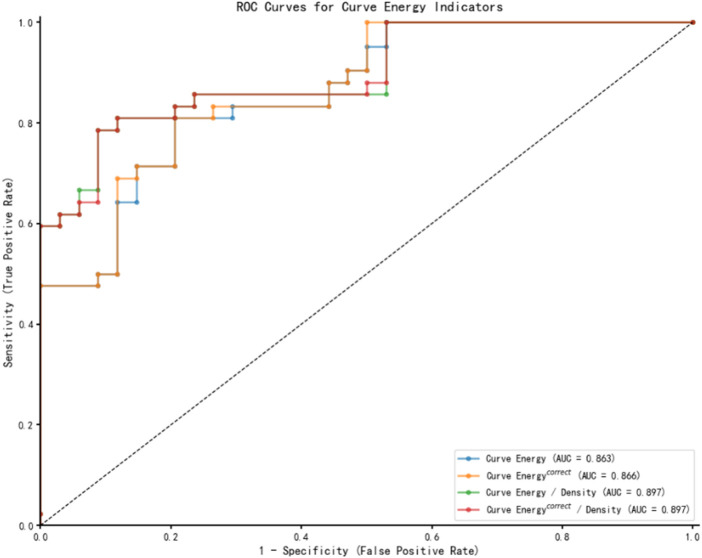
ROC curves differentiate the *Demodex* blepharitis group from the normal control group.

Following the multivariate regression analysis, Receiver Operating Characteristic (ROC) curves were constructed to evaluate and compare the diagnostic capabilities of various energy indicators for *Demodex* blepharitis, as depicted in Figure 2. Table 4 presents the area under the curve (AUC) values along with the sensitivity and specificity analyses. The ROC results indicate that the energy curve-related indices exhibit high diagnostic accuracy and a strong ability to distinguish the *Demodex* group from the control group. Notably, the two density-adjusted indices, Curve Energy/Density and Curve Energy ^correct^/Density, achieve a sensitivity of 78.6% (95% CI: 69.3%–87.8%) and a specificity of 91.2% (95% CI: 84.8%–97.6%) at the optimal threshold. With an AUC of 0.897 (95% CI: 0.829–0.965), they outperform Curve Energy (AUC = 0.863) and Curve Energy ^correct^ (AUC = 0.866), demonstrating effectiveness and reliability in diagnosis.

**TABLE 4:** The AUC and the analysis of sensitivity and specificity.

Energy indices	AUC (95%CI)	Sensitivity (95%CI)	Specificity (95%CI)	Cut-off value	Youden index
Curve Energy	0.863 (0.783∼0.942)	81.0% (72.1%∼89.8%)	79.4% (70.3%∼88.5%)	19.707	0.604
Curve Energy ^correct^	0.866 (0.788∼0.945)	81.0% (72.1%∼89.8%)	79.4% (70.3%∼88.5%)	19.315	0.604
Curve Energy/Density	0.897 (0.829∼0.965)	78.6% (69.3%∼87.8%)	91.2% (84.8%∼97.6%)	85.173	0.698
Curve Energy ^correct^/Density	0.897 (0.829∼0.965)	78.6% (69.3%∼87.8%)	91.2% (84.8%∼97.6%)	82.797	0.698

## 4 Discussion

*Demodex* infestation induces a spectrum of ocular pathologies, including lash folliculitis, glandular duct distortion, and focal meibocyte apoptosis, collectively contributing to destabilization of the ocular surface. Current evidence establishes *Demodex* as an etiological factor in various anterior segment disorders, including chronic blepharitis, conjunctivitis, and MGD (7, 21). Although previous studies have identified blepharitis as a potential risk factor for MG damage, the specific morphological alterations associated with *Demodex* blepharitis remain poorly characterized. Current literature predominantly focuses on inflammatory markers and clinical symptoms, with limited data quantifying glandular structural changes through advanced imaging modalities (Deng et al., 2024; Sun et al., 2022)

In this study, we recruited patients with or without *Demodex* infestation, utilizing a quantitative analysis system powered by AI to evaluate and compare the morphological alterations of MGs. The relationship between ocular surface examination and MG morphology was also analyzed. Our study found that compared to the control group, the *Demodex* group exhibited significantly lower NIBUT. The shortened non-invasive TBUT indicates compromised tear film stability and heightened ocular surface vulnerability, making the ocular surface more susceptible to external stimuli, which can cause discomfort. No statistically significant variation was observed in TMH, CFS and OSDI between the *Demodex* group and the normal control group. However, previous studies verified that the ocular discomfort and OSDI score were significantly higher in *Demodex* infested MGD patients, compared with *Demodex*-negative MGD patients. It is speculated that *Demodex* infection may be an important factor in causing or exacerbating damage to the ocular surface and MGs (Yan et al., 2020; Pan and Chen, 2021). Liu et al. (2022) have reported that the ocular *Demodex*-positive group demonstrated a significant elevation in OSDI scores, meibum quality and tear film instability compared to the ocular *Demodex*-free group. Our results do not fully align with those reported in their studies. We surmised that the probable cause was that our subjects represented earlier-stage disease where clinical signs are subtler, and this aligns with Sun et al. ’s finding that symptom severity correlates with patient age (Sun et al., 2022).

In terms of MG morphology, we found that the changes of the MG morphology in the upper eyelid are more substantially connected with MGD than those in the lower eyelid in our previous study, as other previous studies have shown that the upper eyelid provides clearer and higher-quality images compared to the lower eyelid, minimizing uneven focus and demonstrating stronger correlations with clinical indicators (Deng et al., 2021; Swiderska et al., 2022). Therefore, this study only recruited participants with upper eyelid involvement. In this study, the lid margin score and meiboscore were significantly higher in the *Demodex* group than in the normal control group. These findings align with prior studies reporting elevated lid margin inflammation and gland atrophy in *Demodex* blepharitis which is attributed to mite-induced mechanical damage and bacterial vector effects (Yan et al., 2020; Liu et al., 2022). However, conventional metrics such as MG height, width, tortuosity, and density have not adequately captured the overall features of uneven MG morphological changes in prior research (Zhang et al., 2022; Wei et al., 2024). As Arita et al. (2014) demonstrated, these parameters only assess static gland attributes, not distribution patterns, where central glands show significant shortening without changes in width (Wei et al., 2024). Similarly, Zhang et al. (2022) demonstrated that global MG density correlates strongly with MGD severity but may overlook regional pathology patterns. It was observed that the distribution of MG dropout exhibits increased unevenness among patients affected by *Demodex* mite infestation. Several parameters are introduced to measure data discreteness, such as variance, standard deviation, or the total variation-based uneven index (Liu et al., 2022; Yu et al., 2022). The Uneven index based on total variation, is a novel index to evaluate the overall features of MG atrophy, which refers to the addition of the absolute value of the difference in the height of each adjacent pair of glands in the meibography images and can be used as a quantitative measure of the unevenness of MG atrophy. However, the computational complexity of this metric limits its practical utility in time-constrained clinical settings.

To address these limitations, we introduced the index of AI based Energy Curve in a different part of meibography. Several AI models for MG analysis (Li et al., 2024) have already been proposed. Although ResNet-based methods (Saha et al., 2022) excel at meiboscore grading, they cannot quantify spatial heterogeneity - the key innovation of our metric. This capability echoes Khan et al.'s GAN approach (Khan et al., 2021) for boundary enhancement but provides clinically actionable quantification. The energy curve quantifies uneven atrophy by using the two-dimensional curvature energy of the glandular margin, surpassing the limitations of traditional static metrics such as length and width. It enables rapid, non-invasive, and equipment-agnostic application, but it also imposes stringent requirements on the quality of meibography images. The analysis involves three types of metrics: the raw curve energy value, the tarsal margin corrected value, and the density normalized energy indices. The raw value directly quantifies the absolute curvature of the MG margin but is confounded by the intrinsic morphology of the tarsal. This interference can be mitigated by correcting the tarsal margin. Dividing these indices by gland density further removes scale effects arising from variable gland counts, yielding more reliable results.

In this study, a statistically significant difference was observed in energy curve indices between the *Demodex* group and the normal control, with the uneven atrophy in the *Demodex* group being more severe than that in the normal control. This finding left us with one hint that the Energy Curve could distinguish between *Demodex* infestation patients and DED patients. We hypothesized that the observed relationship may be explained by two distinct pathways: (Rabensteiner et al., 2019): *Demodex* infestation can directly damage MGs through mechanical obstruction and inflammatory cascades, leading to measurable morphological changes; (Elston and Elston, 2014); pre-existing gland dysfunction may create a favorable microenvironment for mite proliferation. This bidirectional relationship highlights the importance of comprehensive clinical evaluation, rather than relying solely on isolated parameters for diagnosis.

In current practice, identifying *Demodex* infections typically relies on clinical symptoms and the presence of cylindrical dandruff on eyelashes. Subsequently, confirmation of *Demodex* infestation is achieved through positive findings from light microscopic examination of eyelashes or confocal microscopy. Sometimes we do not pay attention to the condition of eyelashes, and only do a dry eye examination, which will easily lead to a missed diagnosis. Meibography, a non-invasive imaging technique, enables real-time visualization of MG morphology and is widely integrated into dry eye diagnostic workflows. We are the first to utilize meibography to calculate the Energy Curve to evaluate the morphological unevenness of MGs. However, we found that the calculation result of the Energy Curve may be influenced by the MG density. We attempted to use MG density to correct the Energy Curve. Notably, the normalized Energy Curve index demonstrated robust diagnostic performance (AUC = 0.897), highlighting its potential for clinical implementation. Its simplified visualization facilitates rapid screening by non-specialists and also aids clinicians in making more accurate diagnostic and treatment decisions. By identifying irregular atrophy of the MGs, the energy curve can provide guidance for targeted interventions and monitor treatment efficacy over time. However, it is worth noting that similar MG morphological changes can occasionally be caused by other diseases in clinical practice. Therefore, this index should be used as a supplementary tool to direct *Demodex* detection or additional clinical assessments, rather than a replacement. Combining the energy curve biomarker with other diagnostic tools can enhance diagnostic specificity and provide a more comprehensive evaluation.

There were several limitations in this study. First, we did not include the parameters from the lower eyelid, which may introduce selection bias given known topographic differences in MG morphology between eyelids. Future research should expand morphological assessments to include lower eyelid meibography, addressing potential topographic variability in gland dropout patterns. Concurrently, we will expand the sample size by including more subjects to enhance the representativeness and generalizability of the study results. Second, under the present inspection conditions, we can not precisely count the number of *Demodex* mites within each eyelash follicle. Therefore, we were unable to determine the relationship between glandular atrophy and *Demodex* quantity in individual glands. Future investigations should focus on elucidating the relationship between *Demodex* mite burden and both clinical manifestations and structural alterations in MG architecture, particularly in early-stage cohorts. Lastly, the cross-sectional nature of this study limits its ability to establish causal relationships. Future longitudinal studies could provide more robust evidence by tracking changes in MG parameters and clinical symptoms over time in a cohort of patients with *Demodex* blepharitis. Furthermore, we will also explore more refined representations of edge energy. For example, we will integrate multi - scale edge information, enhance sensitivity to curvature, and design adaptive weighting mechanisms for complex boundary shapes. This will further improve the accuracy of quantitative analysis and its practical value in clinical applications.

## 5 Conclusion

To quantify the irregular atrophy of MGs caused by *Demodex* infestation, we developed the energy curve metric. As a non-invasive biomarker, it’s rapid and easy to perform, making it suitable for early diagnosis, especially in settings with limited resources. The introduction of the energy curve also offers a novel approach for future research into the mechanisms of MG uneven atrophy.

## Data Availability

The raw data supporting the conclusions of this article will be made available by the authors, without undue reservation.

## References

[B1] Akkucuk S. Kaya O. M. Aslan L. Ozdemir T. Uslu U. (2023). Prevalence of Demodex folliculorum and Demodex brevis in patients with blepharitis and chalazion. Int. Ophthalmol. 43 (4), 1249–1259. 10.1007/s10792-022-02523-y 36255613

[B2] Arita R. Suehiro J. Haraguchi T. Shirakawa R. Tokoro H. Amano S. (2014). Objective image analysis of the meibomian gland area. Br. J. Ophthalmol. 98 (6), 746–755. 10.1136/bjophthalmol-2012-303014 23813417 PMC4033206

[B3] Bitton E. Aumond S. (2021). Demodex and eye disease: a review. Clin. Exp. Optom. 104 (3), 285–294. 10.1111/cxo.13123 32885484

[B4] Branch ADEAC (2018). Expert consensus on diagnosis and treatment of demodex blepharitis in China. Zhonghua Yan Ke Za Zhi. 54 (7), 491–495. 10.3760/cma.j.issn.0412-4081.2018.07.004

[B5] Cheng A. M. Sheha H. Tseng S. C. (2015). Recent advances on ocular demodex infestation. Curr. Opin. Ophthalmol. 26 (4), 295–300. 10.1097/ICU.0000000000000168 26058028

[B6] Dai Ql X. Lin X. Fu Y. Ye J. (2021). A novel Meibomian gland morphology analytic system based on a convolutional neural network. IEEE Access (99), 1.

[B7] Deng X. Qi W. Zhao S. Yang R. Zhang C. Huang Y. (2024). Effects of climate factors and demodex infestation on Meibomian gland dysfunction-associated dry eye diseases. Sci. Rep. 14 (1), 284. 10.1038/s41598-023-50858-y 38168639 PMC10762231

[B8] Deng Y. Wang Q. Luo Z. Li S. Wang B. Zhong J. (2021). Quantitative analysis of morphological and functional features in meibography for meibomian gland dysfunction: diagnosis and grading. EClinicalMedicine 40, 101132. 10.1016/j.eclinm.2021.101132 34541482 PMC8435692

[B9] Ding S. C. Su J. J. Zhan Q. Wang J. M. Zheng F. Fang X. X. (2024). Factors affecting Meibomian gland area loss in symptomatic adults. Int. J. Ophthalmol. 17 (6), 1036–1041. 10.18240/ijo.2024.06.07 38895686 PMC11144782

[B10] Elston C. A. Elston D. M. (2014). Demodex mites. Clin. Dermatol. 32 (6), 739–743. 10.1016/j.clindermatol.2014.02.012 25441466

[B11] Khan Z. K. Umar A. I. Shirazi S. H. Rasheed A. Qadir A. Gul S. (2021). Image based analysis of Meibomian gland dysfunction using conditional generative adversarial neural network. BMJ Open Ophthalmol. 6 (1), e000436. 10.1136/bmjophth-2020-000436 PMC7883862 33644402

[B12] Kim J. T. Lee S. H. Chun Y. S. Kim J. C. (2011). Tear cytokines and chemokines in patients with demodex blepharitis. Cytokine 53 (1), 94–99. 10.1016/j.cyto.2010.08.009 21050771

[B13] Li L. Xiao K. Shang X. Hu W. Yusufu M. Chen R. (2024). Advances in artificial intelligence for meibomian gland evaluation: a comprehensive review. Surv. Ophthalmol. 69 (6), 945–956. 10.1016/j.survophthal.2024.07.005 39025239

[B14] Liang L. Liu Y. Ding X. Ke H. Chen C. Tseng S. C. G. (2018). Significant correlation between Meibomian gland dysfunction and keratitis in young patients with Demodex brevis infestation. Br. J. Ophthalmol. 102 (8), 1098–1102. 10.1136/bjophthalmol-2017-310302 29055903

[B15] Lin A. Ahmad S. Amescua G. Cheung A. Y. Choi D. S. Jhanji V. (2024). Blepharitis preferred practice pattern®. Ophthalmology 131 (4), P50–P86. 10.1016/j.ophtha.2023.12.036 38349296

[B16] Liu X. Fu Y. Wang D. Huang S. He C. Yu X. (2022). Uneven index: a digital biomarker to prompt demodex blepharitis based on deep learning. Front. Physiol. 13, 934821. 10.3389/fphys.2022.934821 35899029 PMC9309610

[B17] Lu Z. Wu S. Yan C. Chen J. Li Y. (2021). Clinical value of energy spectrum curves of dual-energy computer tomography May help to predict pathological grading of gastric adenocarcinoma. Transl. Cancer Res. 10 (1), 1–9. 10.21037/tcr-20-1269 35116234 PMC8797754

[B18] Pamela J. R. K. Kashyap R. (2016). Energy based methods for medical image segmentation. Int. J. Comput. Appl. 146 (6), 22–27. 10.5120/ijca2016910808

[B19] Pan S. Chen Y. (2021). A clinical study on the correlation between demodex infestation and ocular surface changes in patients with Meibomian gland dysfunction. Indian J. Ophthalmol. 69 (9), 2389–2394. 10.4103/ijo.IJO_3641_20 34427228 PMC8544033

[B20] Rabensteiner D. F. Aminfar H. Boldin I. Nitsche-Resch M. Berisha B. Schwantzer G. (2019). Demodex mite infestation and its associations with tear film and ocular surface parameters in patients with ocular discomfort. Am. J. Ophthalmol. 204, 7–12. 10.1016/j.ajo.2019.03.007 30885709

[B21] Saha R. K. Chowdhury A. M. M. Na K. S. Hwang G. D. Eom Y. Kim J. (2022). Automated quantification of Meibomian gland dropout in infrared meibography using deep learning. Ocul. Surf. 26, 283–294. 10.1016/j.jtos.2022.06.006 35753666

[B22] Sun X. Liu Z. Sun S. Zhao S. Zhang X. Huang Y. (2022). The correlation between demodex infestation and Meibomian gland dysfunction at different ages. BMC Ophthalmol. 22 (1), 388. 10.1186/s12886-022-02610-9 36183062 PMC9526341

[B23] Swiderska K. Read M. L. Blackie C. A. Maldonado-Codina C. Morgan P. B. (2022). Latest developments in meibography: a review. Ocul. Surf. 25, 119–128. 10.1016/j.jtos.2022.06.002 35724917

[B24] Wei J. Xiao K. Cai Q. Lin S. Lin X. Wang Y. (2024). Meibomian gland alterations in allergic conjunctivitis: insights from a novel quantitative analysis algorithm. Front. Cell Dev. Biol. 12, 1518154. 10.3389/fcell.2024.1518154 39834396 PMC11743466

[B25] Yan Y. Yao Q. Lu Y. Shao C. Sun H. Li Y. (2020). Association between demodex infestation and ocular surface microbiota in patients with demodex blepharitis. Front. Med. (Lausanne) 7, 592759. 10.3389/fmed.2020.592759 33251239 PMC7672197

[B26] Yeu E. Koetting C. (2025). Meibomian gland structure and function in patients with demodex blepharitis. J. Cataract. Refract Surg. 51, 359–365. 10.1097/j.jcrs.0000000000001619 39853246

[B27] Yin Y. Gong L. (2015). Uneven meibomian gland dropout over the tarsal plate and its correlation with meibomian gland dysfunction. Cornea. 34 (10), 1200–1205. 10.1097/ICO.0000000000000533 26203752

[B28] Yu X. Fu Y. Lian H. Wang D. Zhang Z. Dai Q. (2022). Uneven meibomian gland dropout in patients with meibomian gland dysfunction and demodex infestation. J. Clin. Med. 11 (17), 5085. 10.3390/jcm11175085 36079014 PMC9457096

[B29] Zhang Z. Lin X. Yu X. Fu Y. Chen X. Yang W. (2022). Meibomian gland density: an effective evaluation index of meibomian gland dysfunction based on deep learning and transfer learning. J. Clin. Med. 11 (9), 2396. 10.3390/jcm11092396 35566522 PMC9099803

